# Exploring the provision and motives behind the adoption of health-promotion programmes in professional football clubs across four European countries

**DOI:** 10.1371/journal.pone.0259458

**Published:** 2021-11-19

**Authors:** Øystein B. Røynesdal, Femke van Nassau, Nai Rui Chng, Hugo Pereira, Eivind Andersen, Christopher Bunn, Judith G. M. Jelsma, Maria W. G. Nijhuis-van der Sanden, Glyn C. Roberts, Marit Sørensen, Irene van de Glind, Theo van Actherberg, Cindy M. Gray

**Affiliations:** 1 Department of Teacher Education, NLA University College, Bergen, Norway; 2 Department of Sport and Social Sciences, Norwegian School of Sport Sciences, Oslo, Norway; 3 Department of Public and Occupational Health, VU University Medical Center, Amsterdam, Netherlands; 4 Institute of Health and Wellbeing, College of Social Sciences, University of Glasgow, Glasgow, United Kingdom; 5 Interdisciplinary Center for the Study of Human Performance (CIPER), Faculty of Human Kinetics, University of Lisbon, Lisbon, Portugal; 6 Research Institute for Health Sciences, IQ Healthcare, Radboud University Medical Center, Nijmegen, The Netherlands; 7 Research and Development, FWG, Utrecht, Netherlands; 8 Department of Public Health and Primary Care, Academic Centre for Nursing and Midwifery, KU Leuven, Leuven, Belgium; Universitat Luzern, SWITZERLAND

## Abstract

This study mapped existing health-promotion provisions targeting adults in professional football clubs across England, the Netherlands, Norway, and Portugal, and explored motives behind the clubs’ adoption of the European Fans in Training (EuroFIT) programme. We surveyed top-tier football clubs in the four countries and interviewed representatives from football clubs and the clubs’ charitable foundation who delivered EuroFIT. The findings showed large between-country differences, with football clubs in England reporting far greater healthy lifestyle provision than other countries. Relatively few health-promotion programmes targeted adults, particularly in the Netherlands, Portugal, and Norway. Club representatives reported that the motives for adopting the EuroFIT programme often involved adhering to both the social objectives of the football club or club’s foundation and business-related objectives. They viewed the scientific evidence and evaluation underpinning EuroFIT as helpful in demonstrating the value and potential future impact of both the programme and the clubs’ wider corporate social responsibility provision.

## Introduction

Health is one of the main domains of corporate social responsibility (CSR) provision emphasised by European professional football clubs [1]. New approaches are necessary to tackle the current state of public health in many European countries, as many adults in those countries do not meet the recommended physical activity levels (150 min of moderate to vigorous physical activities (MVPA) per week) [2] with country differences ranging from 20%–62% [3]. In addition, 24% of European adults are considered obese (BMI < 30) [4], which may lead to an increased prevalence of non-communicable lifestyle-related diseases such as coronary heart disease and Type 2 diabetes [5]. It is particularly difficult to reach and engage men [6, 7], especially those from lower socio-economic groups, in healthy lifestyle interventions [8]. However, it has been argued that professional football organisations can have a positive public health impact by engaging people in health-related programmes or interventions through their CSR provision [9–11].

Contemporary literature supports the promise of football clubs as venues for delivering health-promotion programmes to adults in this way. The Football Fans in Training (FFIT) programme succeeded in helping middle-aged male football fans in Scottish Premier League clubs lose weight and improve their physical activity levels [12], and were successfully extended to German Bundesliga clubs [13]. FFIT was designed to appeal to men in terms of its content and delivery style, while harnessing their loyalty to professional football teams [14]. Building on and extending FFIT, European Fans in Training (EuroFIT) helped overweight male football fans improve their physical activity, diet, weight, vitality, well-being, self-esteem, and biomarkers of cardiometabolic health in 15 professional football clubs in England, the Netherlands, Norway, and Portugal [15]. Other health-promotion interventions in the UK have improved the physical and mental health of adult fans through their associated football clubs in lower-division and the English Premier League [16–19].

Adoption of health-promotion interventions within professional football organisations is a process where designated managers or decision-makers in clubs or their foundations (a charitable arm of football clubs) decide whether they should commit to delivering a specific project [20, 21]. Despite recent encouragement from the Union of European Football Associations (UEFA), to adopt evidence-based community intervention projects [22] and the focus on health as part of clubs’ CSR provision [1, 23], relatively few professional football organisations are adopting such health-promotion programmes in real-world settings [24]. A recent audit of Spanish football clubs reported that club representatives had contrasting views about their respective club’s role in health promotion provision; most viewed health-promoting CSR activities as outside their core business as a football club, while others believed it supported the club’s social role [24]. In their integrative framework of CSR integration in European professional football clubs, Fifka and Jaeger [25] propose a complex process involving both internal (e.g. club values) and external motives (e.g. societal needs/political/institutional incentives) to drive decision-making, but also the necessity of considering the strategic (e.g. core strategy) and operational elements (e.g. infrastructure) of club organisations. Other studies investigating the decision-making processes of managers in community sports trusts (CST) or foundations associated with UK professional football clubs [20, 26] have described that decisions on whether or not to adopt specific programmes or interventions are often underpinned by the potential impact on local society and congruence with the business objectives of the club. Further, decisions are driven by whether internal challenges and the constraints of delivering the programme are manageable, so that the value of a given CSR programme can be harvested to maximise social and business-related impact (see Anagnostopoulos et al. [20] for more detail).

There is growing evidence of the public health impact from programmes run in professional football clubs targeting adults [12, 15] and a call for a more strategic approach to increase the adoption of health interventions in clubs [21]. However, there is little knowledge as to whether football clubs harness the potential of delivering health-promotion programs to this target group [24]. In addition, studies exploring drivers or motives behind CSR provision have mostly focused on how clubs use drivers or motives as incentives to develop an overall CSR platform as part of an organisational structure or through social partnerships such as foundations or CSTs [27–30]. There is, to the best of our knowledge, no studies that have used the real-world delivery of a health-promotion programme to further understand the motives behind adopting such programmes as part of football clubs’ CSR. We therefore sought to explore the extent of which football clubs in the four countries chosen to deliver EuroFIT ran health-promotion programmes as part of their CSR provision, and to use EuroFIT as a case to further understand why clubs chose to adopt the programme. As such, this study aimed to: a) map the prevalence and type of health-promotion programmes targeting adults in professional football clubs across the top-tier divisions in England, the Netherlands, Norway, and Portugal; and b) to explore the motives of top-tier professional football clubs in these four countries to adopt the healthy lifestyle programme EuroFIT.

### The European Fans in Training (EuroFIT) programme

EuroFIT is an evidence- and theory-based, gender-sensitized, healthy lifestyle programme [15]. EuroFIT harnesses the loyalty that many men feel towards the football club they support to instigate positive long-term changes in health behaviours, whilst promoting social partnerships between professional football clubs, their fans, and local communities across Europe.

To inform the EuroFIT programme development and the potential for programme adoption in European countries, an online and telephone survey mapped existing health programmes promoting increased levels of physical activity and healthier diets, reduced sitting time and alcohol consumption, and weight management in European football clubs in 2014–15. Following this, the EuroFIT programme was delivered in fifteen professional football clubs from the Netherlands, UK, Portugal and Norway during the 2015–16 football season as part of a randomized controlled trial (RCT). The program comprised 12-week, in club stadia, 90-minute sessions combining classroom discussions with graded physical activity group sessions led by community or football coaches, as well as one reunion session 6–9 months after completion. Each club received a research intervention package (e.g. recruitment and delivery materials), general support from the local researchers, and full funding from the research project for a full-scale programme delivery in two phases (both intervention and control group delivery). For more details about the EuroFIT programme, please see van Nassau et al. [31] and Wyke et al. [15]).

Following the RCT, local researchers in the research consortium recruited six additional professional football clubs from the same countries to deliver a real-world pilot implementation of EuroFIT during the 2017/18 football season. For this phase, each club received a pilot implementation programme package (e.g. recruitment and some program delivery materials) and informational support (e.g. template for a needs and cost-benefit assessment, stakeholder involvement) from local researchers, but no financial support from the research project for a one-time delivery. Table 1 shows the clubs that delivered EuroFIT programme, their competitive level, and which part of the club that was responsible for coordinating and delivering the programme. In each country, the football club representatives and their coaches were trained by local research teams for both RCT and pilot implementation delivery. The local research teams comprised researchers and research assistants located at domestic universities in Norway, Portugal, Scotland, the Netherlands, and Belgium, as well as a pan-European operational partner focusing on health-promoting environments throughout European football.

**Table 1:** Clubs involved in the EuroFIT RCT or pilot implementation delivery.

Club	Country and league	Organizational structure in charge of EuroFIT delivery	Type of program delivery
ADO den Haag	Netherlands (Eresdivisie)	Charitable foundation/CST	RCT
Groningen	Netherlands (Eresdivisie)	Charitable foundation/CST	RCT
PSV	Netherlands (Eresdivisie)	Charitable foundation/CST	RCT
Vitesse	Netherlands (Eresdivisie)	Charitable foundation/CST	RCT
Heerenveen	Netherlands (Eresdivisie)	Charitable foundation/CST	Pilot implementation
Porto	Portugal (La Primeira)	Sport department	RCT
Club Sporting	Portugal (La Primeira)	Sport department	RCT
Benfica	Portugal (La Primeira)	Charitable foundation/CST	RCT
SC Braga	Portugal (La Primeira)	Charitable foundation/CST	Pilot implementation
FC Rio Ave	Portugal (La Primeira)	Medical department	Pilot implementation
Vålerenga FK	Norway (Eliteserien)	Charitable foundation/CST	RCT
SK Brann	Norway (Eliteserien)	CSR and Sport department	Pilot implementation
Rosenborg BK	Norway (Eliteserien)	CSR and Sport department	RCT
Leicester	UK (Premier League)	Charitable foundation/CST	Pilot implementation
Blackburn	UK (Championship)	Charitable foundation/CST	Pilot implementation
Arsenal	UK (Premier League)	Charitable foundation/CST	RCT
Man City	UK (Premier League)	Charitable foundation/CST	RCT
Newcastle	UK (Premier League)	Charitable foundation/CST	RCT
Stoke	UK (Premier League)	Charitable foundation/CST	RCT
Everton	UK (Premier League)	Charitable foundation/CST	RCT

## Methods

### Design

This study consisted of an online survey of top-tier professional football clubs in England, the Netherlands, Norway, and Portugal, with targeted follow-up telephone surveys of non-responders in a sample of clubs, and exploratory semi-structured interviews with representatives from clubs who delivered the EuroFIT programme during the RCT and the following real-world pilot implementation.

**Fig 1:**
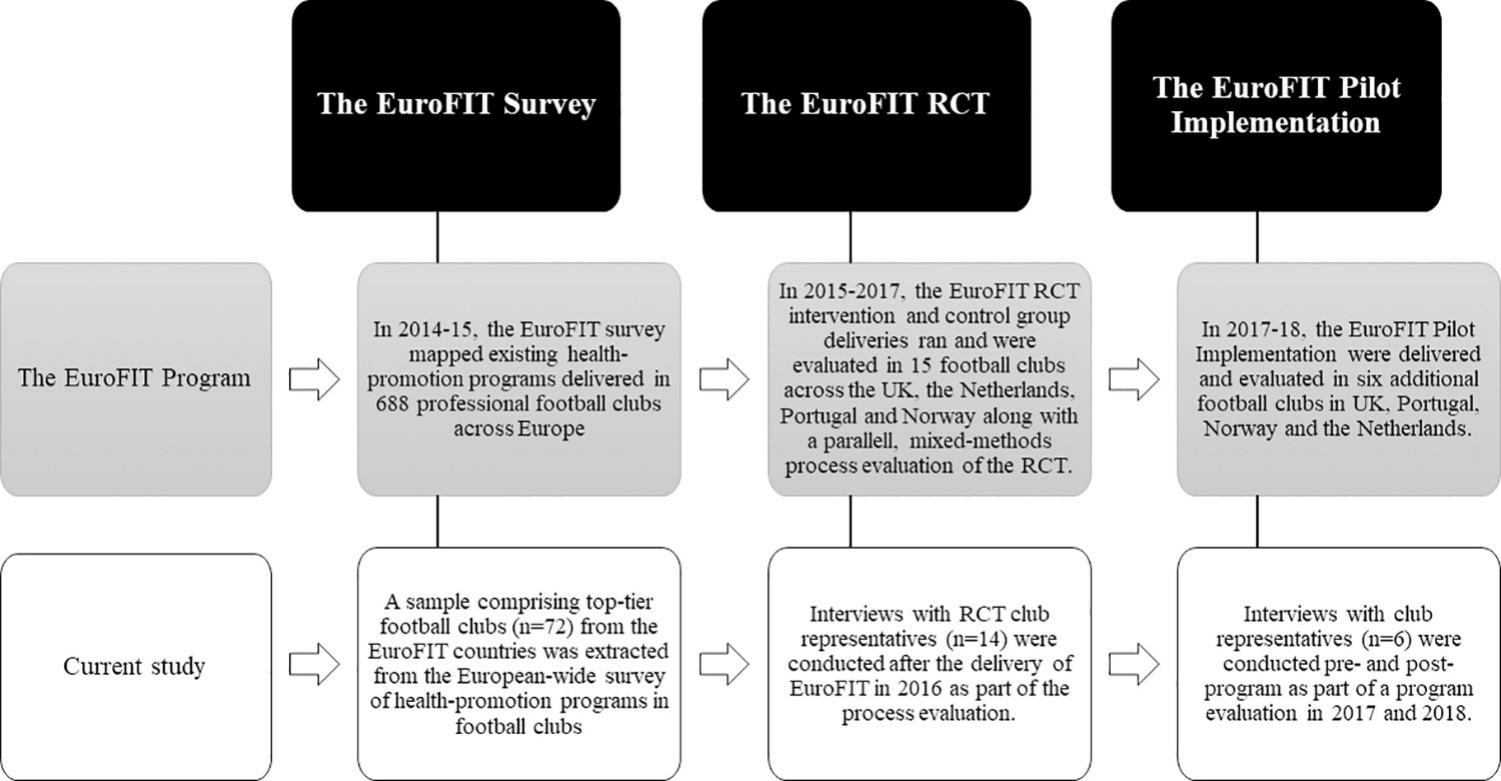
Timeline and overall process of the EuroFIT program and the data used for this current study.

Fig 1 illustrates the overall process of the EuroFIT program, and the data used for this study. We extracted a sample comprising top-tier football clubs from the EuroFIT countries from the European-wide survey of health-promotion programs in football clubs administered prior to the RCT. The interviews were part of the process evaluation that ran parallel to the RCT intervention and the pilot implementation of the EuroFIT programme [31, 32]. The combined data enabled us to generate a fuller understanding of health-promotion programmes in top-tier football clubs across the four countries [33].

### Data collection procedure and participants

Ethical approval for the study was obtained from country-specific ethics committees for the two data collections at different time points: (Ethics committee of the VU University Medical Centre [2014/276] and [2015/184]; Regional Committees for Medical and Health Research Ethics, Norway [38900/3/LT] and [2015/1862]; Ethics Council of the Faculty of Human Kinetics, University of Lisbon [20/2014] and Lisbon [CEFMH 36/2015], and Ethics Committee at the University of Glasgow College of Social Sciences [400130205] and Ethics Committee at the University of Glasgow College of Medicine, Veterinary and Life Sciences [UK] [200140174]).

All survey respondents consented in writing or orally before undertaking the online or telephone survey. All interviewees provided written informed consent before participating in the qualitative data collection.

#### Online and telephone survey

The online survey was distributed via Survey Monkey on 19 February 2014 to individual club representatives working in CSR or related departments or foundations in 688 professional football clubs in the top division of 52 UEFA member nations. We designed the survey specifically to inform the development of the EuroFIT programme and to understand the potential for programme adoption in each country. The survey included 26 closed questions to capture information on physical activity, healthy eating, and weight management initiatives in which the clubs were engaged (e.g. Does your club run projects promoting active lifestyles and participation in sports to fans and the public?), as well as groups targeted by the types of programmes delivered (young people or adults). As EuroFIT is a physical activity programme targeting adult men, the target groups for physical activity programmes were further broken down to children (6–11 years old), youth (11–17 years old), men (<17 years old), and women (<17 years old) to understand the potential for adoption of EuroFIT. As weight management was not a primary target of the EuroFIT programme, we used an open question to elicit further relevant information on specified target groups for these programmes.

Following three reminders to complete the online survey, local members of the research group contacted 20 non-responding football clubs who had responded to, but not fully completed, the online survey by telephone. The telephone survey comprised a selection of 13 questions used in the online survey (e.g. Does your club promote healthier diet through fan or public engagement projects?) that were considered to generate a good understanding of the club context in the respective countries.

Due to the very low overall survey response rate from European football clubs, and as we delivered the EuroFIT programme in England, Norway, the Netherlands, and Portugal and only interviewed club representatives in these countries, we chose to include only the survey results from top-tier football clubs (n = 72) in these countries for this study.

#### Individual interviews

Twenty male and female EuroFIT club representatives aged between 24 and 57 years participated in the interviews. We contacted and purposively sampled designated club representatives who were personally involved in the decision-making regarding the EuroFIT programme, regardless of their role at the club (e.g. football school executive coordinator, charitable foundation project manager, CSR director) [34]. We conducted face-to-face or telephone interviews with 14 representatives from the RCT clubs between March and June 2016 after programme delivery, and 6 representatives from real-world pilot implementation clubs pre- and post-programme delivery between January and October 2017.

At least six weeks prior to data collection, a local researcher notified the club representatives of the nature and purpose of the interview. Researchers from local research teams conducted interviews in the local language. We prepared a semi-structured interview guide to elicit open-ended responses. Separate interview guides were prepared for the RCT and pilot implementation clubs because they received different programme packages, funding, and support during the preparatory phase. The RCT club interviews ranged in duration from 18 to 79 minutes, whereas interviews with implementation clubs lasted between 15 and 59 minutes. All interviews were recorded using a digital audio recorder, assigned an ID tag as an RCT (e.g. RCTClub103_Country) or pilot implementation club (e.g. PilotClub301_Country) and transcribed verbatim in the local language by either a professional transcription company or a local research assistant. The transcribed data were stored securely according to the internal institutional regulations of each country.

### Analysis

#### Online and telephone survey

Responses from the online and telephone surveys were exported from Survey Monkey, imported into Microsoft Excel 2016, and combined into one dataset to check for any duplicate responses. We analysed the survey data using descriptive statistics on a country-to-country basis.

Some respondents did not fully answer all the questions. If a respondent did not answer a closed question or did not answer any of the questions at all, then we treated these values as missing. In the case of open questions, we treated these values as missing if respondents did not provide any specification about weight management target groups or did not answer. All percentage scores are based on the number of clubs that responded directly to a specific question.

#### Individual interviews

Using structured, inductive thematic analysis [32], the first step of the qualitative analysis involved familiarisation of interview transcripts from RCT and pilot implementation clubs in the local language after all programme delivery in clubs had ended. Each local researcher then generated initial codes for all text segments across the complete dataset that included both RCT and pilot implementation clubs. All codes were assigned in English. Each researcher forwarded country-specific codes from these interviews, with exemplary quotes to the first author, who summarised and compiled a preliminary codebook.

Through critical discussion in telephone meetings, we merged codes with topical commonality into groups and discarded duplicate or irrelevant codes. All coding decisions were made with consensus in the research group throughout the analysis. Afterward, the first author put together a consolidated codebook (S1 File) and circulated this to local researchers for review. Only minor clarifications were requested by the researchers (e.g. adding nuances to the phrasing of a code description). An experienced qualitative researcher with knowledge of the data, who did not partake in the initial coding, reviewed the codebook for overlapping code descriptions as a ‘critical friend’ [35].

We adjusted the already-coded transcripts according to the consolidated codebook and coded the remaining interviews. Following a standard template in English, written reports from each country, summarising the coded data descriptively and highlighting differences between RCT and pilot implementation clubs, were sent to the first author. The first author analysed the reports, focusing on the recurrence of codes and themes across countries using a simplified convergence matrix (S2 File) [36]. Lastly, the first author drafted the overall qualitative findings highlighting differences in generated themes between countries before circulating the findings to the co-authors for a critical review of interpretation and feedback.

## Results

### Existing health-promotion programmes in professional football clubs

Of the 72 survey requests sent, 49 football clubs (63.9%) took part in the online or telephone survey mapping current health-promotion programmes. Of those clubs, 29 (59.2%) responded to the online survey, and 20 (40.8%) took part in the telephone survey. However, three did not answer any of the survey questions. Of the remaining 46 respondents, 17 (36.7%) were from England, 12 (26.1%) from Norway, 9 (19.6%) from the Netherlands, and 8 (17.4%) from Portugal. Of the 72 survey requests, 17 (23%) clubs responded in England (out of 20 top tier clubs), 12 (16.7%) (out of 16 clubs) responded in Norway, 9 (12.5%) (out of 18 clubs) in Netherlands and 8 (11.1%) (out of 18 clubs) in Portugal.

#### Physical activity or football programmes in the football clubs

Of the 46 survey respondents, 91.3% indicated that their club provided football or physical activity programmes targeting different populations. As Table 2 shows, 35 (76.1%) respondents reported delivering a physical activity or football-related programme for children, whereas 32 (69.5%) offered similar programmes to young people. Seventeen (37%) clubs offered programmes to men or women only, and 15 (32.6%) clubs to mixed groups of adults (all aged over 17 years). Most football or physical activity programmes for adults are delivered by clubs in the UK. Four (8.7%) clubs delivered no physical activity programmes to any target group across the four countries.

**Table 2:** Frequency of physical activity programs delivered in professional football clubs in four European countries.

		% of total N (n = 46)	England	Norway	Netherlands	Portugal
Children’s (5–11 years) football/physical activity		76.1 (35)	45.7 (16)	17.1 (6)	25.7 (9)	11.4 (4)
Young people’s (11–16 years) football/physical activity		69.5 (32)	50 (16)	25 (8)	15.6 (5)	9.4 (3)
Men football/physical activity		37 (17)	70.6 (12)	17.6 (3)	5.9 (1)	5.9 (1)
Women football/physical activity		37 (17)	64.8 (11)	17.6 (3)	11.8 (2)	5.9 (1)
Men and women football/physical activity		32.6 (15)	66.7 (10)	13.3 (2)	13.3 (2)	6.7 (1)
Reducing levels of prolonged inactivity		26.1 (12)	58.3 (7)	16.7 (2)	16.7 (2)	8.3 (1)
No programme of this type		18.7 (4)	0	50 (2)	0	50 (2)

#### Healthy eating programmes in the football clubs

Thirty-six (73.5%) clubs responded to the question on healthy eating programmes. As Table 3 illustrates, 24 (66.7%) respondents indicated that their club provided healthy eating/food preparation programmes to young people, whereas 13 (36.1%) clubs provided such a programme to adults. Twelve (33.3%) clubs provided no healthy eating programmes. Almost all of the clubs who reported delivering healthy eating programmes were English. Ten football clubs (21.7%) did not answer this question.

**Table 3:** Frequency of healthy eating programmes delivered in professional football clubs in four European countries.

	% of total N (n = 36*)	England	Norway	Netherlands	Portugal
Healthier eating / food preparation programmes for young people	66.7 (24)	62.5 (15)	8.3 (2)	20.8 (5)	8.3 (2)
Healthier eating / food preparation programmes for adults	36.1 (13)	76.9 (10)	7.7 (1)	7,7 (1)	7.7 (1)
No programme of this type	33.3 (12)	8.3 (1)	83.3 (10)	8.3 (1)	0 (0)

* Missing values from 10 clubs not included.

#### Weight management programmes in the football clubs

Of the 46 survey respondents, 38 clubs (82.6%) responded to the question on weight management programmes. As Table 4 illustrates, 28 clubs (73.7%) did not deliver any weight management programmes. All of the clubs who reported having weight management programmes targeting young people or adults were English. Nine additional clubs reported delivering weight management programmes but did not specify the target audience and were excluded.

**Table 4:** Frequency of weight management programmes delivered in professional football clubs in four European countries.

	% of total N (n = 37**)	England	Netherlands	Portugal	Norway
Weight-loss or management programmes for young people	13.5 (5)	100 (5)*	0*	0	0
Weight-loss or management programmes for adults	24.3 (9)	100 (9)*	0*	0	0
No weight-loss or management programmes	75.7 (28)	10.7 (3)	21.4 (6)	28.6 (8)	39.3 (11)

*Clubs either reported delivering no weight management program or did not specify whom they targeted with their program.

**Missing values from 9 clubs not included.

#### Alcohol programmes in the football clubs

A total of 43 (93.5%) football clubs responded to the question on programmes targeting alcohol consumption. As Table 5 shows, five (11.6%) respondents indicated that they promote responsible alcohol drinking on match days, whereas ten (23.3%) reported delivering alcohol reduction programmes to adults at their club. Of these ten clubs, seven (70%) were English. Of the 46 respondents, 30 (69.8%) did not provide this type of programme.

**Table 5:** Frequency of alcohol programmes delivered by professional football clubs in four European countries.

	% of total N (n = 44*)	England	Norway	Netherlands	Portugal
Promotion of responsible drinking to fans on match days	11.4 (5)	100 (5)	0 (0)	0 (0)	0 (0)
Programmes about responsible drinking and the dangers of alcohol	22.7 (10)	70 (7)	10 (1)	10 (1)	10 (1)
No programme of this type	68.2 (30)	16.7 (5)	36.7 (11)	26.7 (8)	20 (6)

*Missing values from 2 clubs not included.

### Adoption of the EuroFIT programme

**Fig 2:**
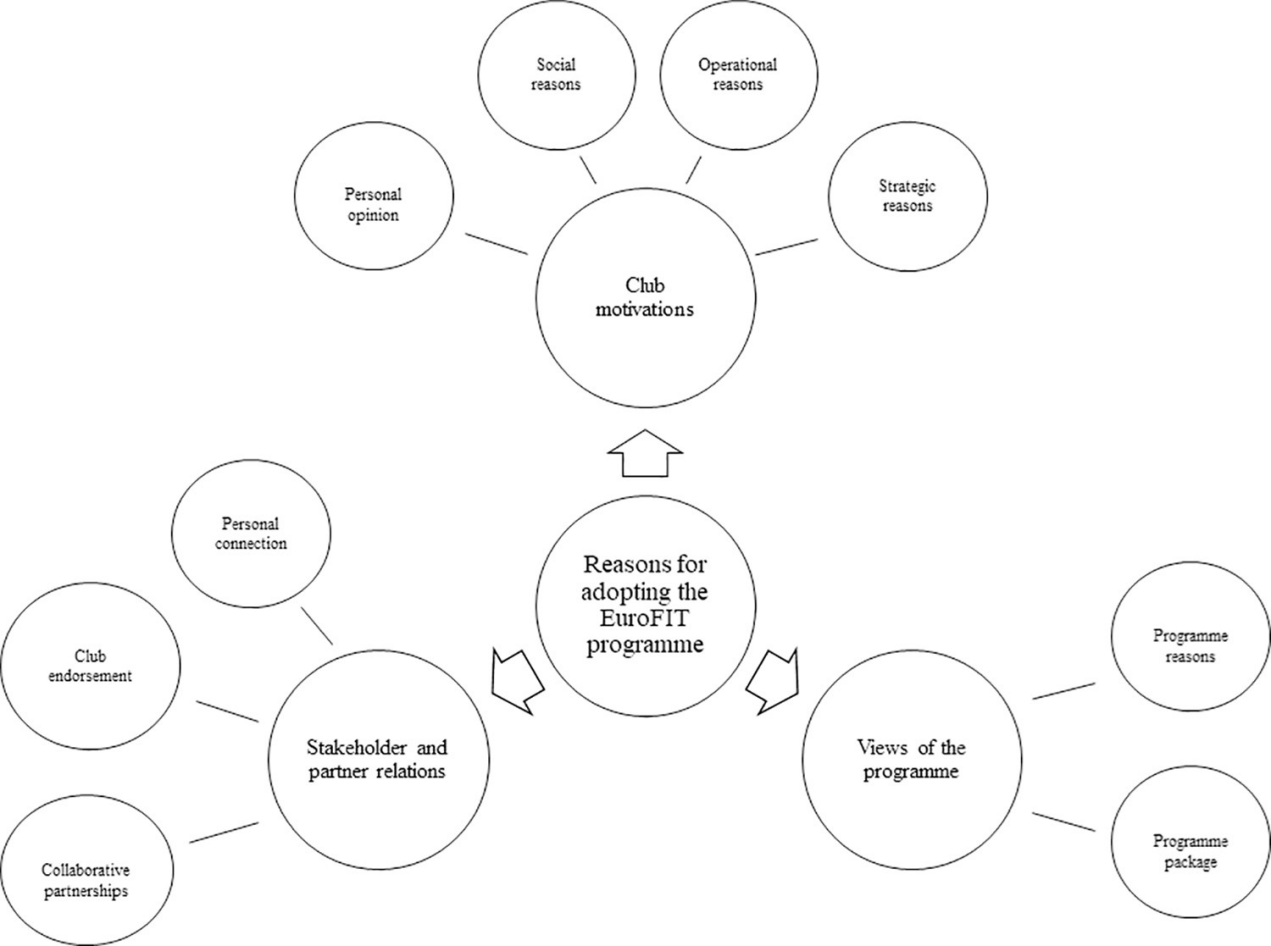
Main themes and sub-themes illustrating motives for adopting the EuroFIT programme.

A total of 21 professional football clubs consented to adopt the EuroFIT programme as part of an RCT or a subsequent real-world pilot implementation. As Fig 2 illustrates, qualitative analysis of interviews with twenty club representatives generated three main themes as to why clubs made this decision: view of the EuroFIT programme, club motivations, and stakeholder and partner relations.

#### View of EuroFIT programme

*Programme reasons*. This refers to how club representatives perceived aspects of the programme as influential in adopting EuroFIT. This includes the programme’s credibility and novelty, the current target population, and potential expansion into other target groups.

RCT clubs in Norway, Portugal, the Netherlands, and UK pilot implementation club representatives emphasised EuroFIT’s credibility as an important reason for its adoption. They liked that it was part of a larger research project aimed at evaluating effectiveness and the scientific evidence underpinning the programme:

… it was really the evidence behind it. We wanted to see what worked, have a go, give it the pilot, and then get that evidence back so then, like I say, when we go to run a larger project and do it on a larger scale, we have that evidence, we know … what does and doesn’t work (PilotClub102_UK).

Pilot implementation clubs in the UK highlighted the fact that other clubs had already delivered EuroFIT (during the RCT) as a contributing factor in increasing programme credibility.

RCT and pilot implementation club representatives from the Netherlands, Portugal, and Norway liked the fact that the programme was something new and that it targeted a new group of their fan base (overweight men). A Portuguese representative said, ‘None of the clubs (in the first division) [have] offered a programme like this to its fans before (RCTClub413_PT)’. None of the UK RCT or pilot implementation clubs mentioned the novelty of the programme or target group as motives.

Another reason for joining expressed by RCT interviewees in the UK, Portugal, and Norway was a potential continuation of the programme at the club after the trial or an expansion of the programme to other target groups, such as women, the elderly, and youth, post-trial:

I remember that we [thought], in the beginning, when introduced to that fact that it should be this and this target group, overweight [men] and that sort of thing … if this is successful, then perhaps we can offer it to different target groups, especially a project in terms of following overweight youth (RCTClub312_NOR).

None of the pilot implementation clubs in any of the four countries or the RCT clubs in the Netherlands referred to the continuation of the programme as a motive for adopting EuroFIT.

*Programme package*. This refers to mentions of how the material or support offered (as described in the EuroFIT programme section) was a contributing or decisive factor when deciding to adopt EuroFIT.

Representatives from RCT and pilot implementation clubs in Norway, Portugal, and the Netherlands discussed how obtaining funding prior to starting new projects was a decisive factor. None of the UK RCTs or pilot implementation clubs spoke of this. A Portuguese RCT club representative said, ‘because the club is not able to make big investments, we would do the project as long as it was cost-free (RCTClub414_PT)’. A Norwegian pilot implementation club representative who had to apply for funding said that it would have been easier to adopt EuroFIT if funding had been offered as part of the programme package.

A representative from a Dutch RCT club explained how clarifying the associated budget expectations and requirements for the club was a decisive factor when deciding whether to deliver EuroFIT:

We have a board and director of the club–so if we have a new project, we propose it to them, and give an explanation and provide them with an overview of the costs, as well as what we get in return (RCTClub206_NED).

Representatives from pilot implementation clubs in the Netherlands and the UK expressed that the comprehensiveness of the program package (including materials and information provided) offered was attractive: ‘I felt that the training and support that was being offered to staff was very good, because we don’t have any health professionals’ (PilotClub2_ UK).

#### Club motivations

*Social reasons*. Social reasons involved clubs viewing EuroFIT as a way to give back to their fans and make a difference in the local community as part of a wider public health agenda in their CSR provision. Targeting both their supporters and people in the wider community appeared to be important for both RCT and pilot implementation clubs in all countries when deciding to adopt the EuroFIT program. A UK RCT club representative explained that, ‘… for me it’s a no-brainer … it’s a men’s health project and as I say seventy-five, maybe seventy per cent of our fan base probably fit the criteria (RCTClub103_UK).

A Portuguese RCT club representative commented on the club’s social role in terms of making a public health impact in the local community, ‘I think the program is very positive …it brings the club to the society, helping fans to have a better quality of life, to exercise and eating [more healthily] (RCTClub413_ PT)’.

*Strategic reasons*. Strategic reasons refer to how the program complied with the club’s social or business-related philosophy and objectives. This included how the club representatives used EuroFIT to promote CSR provision internally to generate support for current or future programmes, development of new partnerships, or potential revenue that club representatives viewed as important when deciding to adopt the programme.

Both RCT and pilot implementation clubs in all countries highlighted the importance of coherence with the club’s overall CSR philosophy as a decisive factor in delivering EuroFIT. A Dutch club representative said, ‘… there has to be a fit with the clubs’ policy–we focus on participation, health, and education. Within these domains, we can choose the projects we like (RCTClub206_NED)’.

Both RCT and pilot implementation clubs from each country but Portugal described using EuroFIT to generate internal support within their club for programmes targeting new areas for CSR engagement, such as health improvement. A representative from a UK pilot implementation club said, ‘It was a good way, like I said, for us to test the water with whether we could make a difference to health, and in this case men’s health (PilotClub102_UK).’ A Dutch RCT club representative explained how they could strategically use the robust evaluation of the EuroFIT programme to demonstrate the value of their charitable work to the partner club:

. . . . As a foundation, it is quite difficult to show what the effect of your program is [within the club]. With EuroFIT it was the perfect opportunity because a scientific evaluation was linked to it. So that is actually the reason (RCTClub209_NED).

Norwegian and UK pilot implementation club representatives talked about using the prospect of adopting the evidence-based EuroFIT programme to expand their CSR provision and policy to include health-promotion:

We simply had to get the buy-in from the senior management and the board [first], and then to push that out to all employees … and all the time we had EuroFIT in mind while developing it (the CSR policy) (PilotClub101_NOR).

RCT and pilot implementation club representatives from most countries, except a pilot implementation club and a RCT club in Norway, discussed marketing or PR as a motive for joining the EuroFIT project. They believed that the programme could draw positive publicity or attract new partners or sponsors to generate a positive club image. A Portuguese RCT club representative explained, ‘… the return on the investment is the club image, to have the fans looking at the club as a club that is close to its fan base (RCTClub415_PT)’. However, one Norwegian RCT club representative did not see PR possibilities as a contributing factor when deciding to adopt programmes, ‘it is sort of just a positive effect of it (the programme), if we also get some publicity cases that are positive (for the club) (RCTClub312_NOR).’

*Operational reasons*. Operational reasons involve aspects of club operations in terms of their experience of running similar programmes, staff capacity, and infrastructure that club representatives viewed as important when deciding to adopt the programme. RCT club representatives from the UK and one RCT club representative from Norway discussed how having previously run successful healthy lifestyle programmes at their club was an important factor in their decisions to deliver the programme. None of the clubs in the Netherlands or Portugal mentioned this aspect as a motive. A Norwegian RCT club representative said:

… I think it was easy for us (to join the programme) because we have worked with these social projects before, so then it wasn’t so difficult to sell it to us… but I think perhaps that it might be more difficult (to adopt the programme) if you have not worked with supporters already, or other target groups (RCTClub312_NOR).

RCT club representatives in Norway, the UK, and Portugal, and Norwegian pilot implementation clubs also discussed the need to clarify whether the club had adequate and available facilities and qualified staff as preconditions for agreeing to adopt the programme. None of the Dutch clubs referred to this motive.

*Personal opinions*. Personal opinions refer to mentions of how foundation/CST or other CSR managers with decision-making authority within the club supported the programme or acted as a catalyst when deciding whether the club should adopt the EuroFIT programme. RCT and pilot implementation club representatives from all countries really believed in the programme and the underpinning concept of helping overweight men to obtain a healthier lifestyle. A UK RCT club representative said, ‘I was, er, championing the cause and wanting to do it right from the word go (RCTClub105_UK).’ Club representatives’ belief in the EuroFIT programme seemed to be a decisive factor: ‘…we (foundation managers) have that role where we can say yes to anything we think is good. So yes, that is why this project fit well (RCTClub208_NED)’.

#### Stakeholders and partners

*Collaborative partnerships*. Collaborative partnerships refer to how support for EuroFIT from significant stakeholders or organisations associated with the club, business partners outside the football club, or collaborations with research institutions were important factors when deciding whether to adopt the programme. Both RCT and pilot implementation club representatives in Norway, and one Dutch RCT club representative spoke of involving their supporter organisations early in the decision-making process:

We put forth a proposal (programme) for our supporter organisation. Those ones are in closest contact with our fans. They knew them really well. We asked, ‘What do you think about this project and would it fit with our male fans?’ They said yes–you should do this (RCTClub206_NED).

A Portuguese RCT club representative talked about potential support from local business partners as a contributing factor:

There is a market for these types of health-related projects. There are health companies that may have interest in being associated to a physical activity programme … I believe other businesses, like clinics with whom we work, could be interested in being close to people (RCTClub413_PT).

RCT club representatives from Portugal, Norway and the UK also talked about how working with the different local universities and researchers contributed to them adopting the program, as they hoped this could evolve into future collaboration: ‘… working with the university as well, as a collaborative partner, [because] it might just be that, from this, we could start other projects together with them’ (RCTClub312_NOR)’.

*Club endorsements*. Club endorsements refer to other football clubs’ positive experiences, support, or endorsements after implementing similar programmes prior to the RCT. For RCT clubs in the UK and the Netherlands, this appeared to be a contributing factor in adopting the programme:

We went to the meeting in England (an information meeting offered to potential clubs interested in delivering EuroFIT) to listen to the experiences of clubs in Scotland, as its inception there, the FFIT (Football Fans in Training) programme. So, based on those experiences we said, yes, this is what we actually should do (RCTClub206_NED).

*Personal connections*. Personal connections refer to how personal relationships between key people within the clubs and an operational partner in the EuroFIT consortium facilitated the decision of whether to adopt the EuroFIT programme. In the UK, several RCT and implementation club representatives discussed meetings and were asked or contacted by an operational partner in the EuroFIT research team before joining. This personal connection was not referred to by either RCT or pilot implementation clubs in the remaining three countries. A UK pilot implementation club representative said:

P: I met [name of person] at a conference in September, just before I’d taken up post here [and] I was aware of the programme because I’d previously worked another [that] football club (PilotClub102_UK).

## Discussion

This study mapped the prevalence and types of health-promotion programmes targeting adults at professional football clubs in four European countries, using delivery of the EuroFIT programme as a case study to understand the motives underlying football clubs’ adoption processes of health-promotion programmes more widely. With this, we highlight ways in which researchers, policymakers and practitioners can harvest the potential of health improvement through professional football by strategically approaching clubs with evidence-based programs.

Our findings correspond with and expand previous studies investigating existing health-promotion programmes [24], overall CSR engagement and integration in football clubs [25, 28, 29, 37] and decision-making processes in foundations associated with professional football clubs in the UK [20, 26]. In light of the integrative framework of CSR in football clubs [25], both RCT and pilot club representatives appeared to understand their club’s adoption process of EuroFIT in a wide context considering both program (e.g., evidence base of program), operational (e.g., club infrastructure and human resources), social (e.g., societal needs) and strategic (e.g., club values and business and social objectives) motives irrespective of existing health-promotion programs and organizational structure. However, there were differences in how some of the Norwegian clubs and Portuguese clubs–of whom had the fewest foundations/CSTs responsible for delivering EuroFIT (see Table 1)–weighted the motives for adopting the program. These clubs discussed operational (e.g. needing adequate human resources and facilities; no experience of health program provision; need for funding) motives more widely compared to the other countries. Contrary to other studies investigating the motives behind CSR engagement in football, there was little emphasis on institutional and political motives to adopt EuroFIT, which is surprising given the focus of health as part of many clubs’ CSR policy [1, 37], European football organizations [22, 23] and the wider political agenda to tackle the public health issues of today [4].

There were also large between-country differences in health-promotion programs across the four countries. In general, adults were an underrepresented target group for health-promotion programmes in football clubs compared to children and youth. These findings also support previous studies on health-promotion programme provision in professional and non-professional football clubs [24, 38]. Relatively few health-promotion programmes targeted adults in the Netherlands, Portugal, and Norway, with football clubs in England reporting far more programmes for adults than the other countries. For example, weight management programs were only reported in the UK. A potential explanation for the difference in programme prevalence could be that one-third of UK professional football clubs now run CSR platforms via partnerships with foundations/CST [30, 37]. These organisational partnerships between the top-tier UK football clubs who delivered EuroFIT and their foundations could result in increased human (e.g. community coaches) and financial resources (e.g. funding), and infrastructure (e.g. facilities) [26], where general CSR engagement in clubs from other countries could be considered an optional activity with few allocated resources [39]. These findings were further substantiated by the narratives of club representatives in Portugal, Norway, and the Netherlands, as they emphasised that EuroFIT targeted a new group in adult men, as well as having little prior experience of running similar health-promotion programmes. Another explanation for the difference in program provision in the UK compared to others, could be that, at the time of enquiry, a few of the UK clubs had already been introduced to the value and impact of health-promotion programs through established professional networks such as the Football Foundation (a football charity funded by the Premier League, the English Football Association FA and the British Government), because of the success of the evidence-based FFIT program in Scotland [12].

As described in the decision-making studies by Anagnostopoulos et al. [20, 26], the RCT and pilot implementation clubs referred to both social and strategic/business-related motives for joining EuroFIT. Club representatives balanced targeting their fans and local community needs to help them make health improvements and adhering to the clubs’ social and business-related policy and outcomes. However, as the social rationale featured strongly in the narratives of both RCT and pilot implementation club representatives, the business-related dimension appeared to vary in strength as a motive to adopt EuroFIT. For example, a Dutch RCT club explained the need to accommodate business-related objectives when undertaking a cost-benefit analysis of delivering EuroFIT to convince senior management of the (positive) impact adopting the programme would have on business-related performance outcomes, whereas a Norwegian RCT club discussed business-related returns, such as a more positive club image or PR, as merely a positive side effect of adopting the programme. These differences could be amplified by the social, cultural, and historical contexts that influence CSR engagement [25, 37], as well as the perceived role of charitable foundations or CSR practices within football clubs, where self-funded foundations might view the returns on business-related objectives differently compared to foundations that depend on the parent club’s resources [39, 40]. However, given the funding offered alongside the choice of delivering the EuroFIT program, this could have allowed more clubs/foundations to weigh the societal impact of targeting adults’ health higher than business-related returns of targeting this group as the largest proportion of many clubs’ fan bases [41].

Considering the narratives obtained in this study and the few existing health-promotion programmes targeting adults across the four countries, it is likely that, in the complex settings where club representatives operate, accommodating both social and strategic/business-related objectives is a pre-requisite that exceed the call for clubs/foundations to target adults’ health as a societal need in their local community. For example, improving the health of adults through the football club’s CSR provision may be considered less impactful [42] or less strategic [43] for business-related purposes then addressing the lifestyle-related needs of young people (children and youths) in the local community. However, as highlighted by both RCT and pilot implementation clubs in this study, adopting an evidence-based programme such as EuroFIT could allow clubs to measure, evaluate, and illustrate the effect of their CSR work to mitigate these issues. Despite this, recent research indicates that evidence-based approaches to health-promotion are rarely adopted in professional football clubs [24] and that very few clubs appropriately evaluate their CSR programmes [24, 44]. However, as discussed by the club representatives, the fact that EuroFIT had a credible evaluation embedded as part of the program contributed positively to programme adoption. It could be that providing the necessary training for coaches or staff to conduct evaluations as part of a wider program package is key in supporting long-term sustainability of EuroFIT in clubs. This could be particularly so in clubs without foundations or that lack human/financial resources designated for CSR provision, where illustrating impact on other key performance indicators (KPIs) beyond the program could be necessary to acquire club resources for adoption or continuation [39, 40].

We contend that adopting evidence-based programmes with embedded evaluations such as EuroFIT or FFIT will help football clubs or associated foundations have a positive impact on their supporters’ and the wider local communities’ health, so they can: a) demonstrate the impact and value of their health-promotion programmes to senior management and the board at their club, and b) generate increased support for CSR provision from key stakeholders both within and outside the club [39, 45]. Combined with the relative paucity of health-promotion programmes targeting adults, this further indicates the potential for other football clubs to adopt existing evidence-based programmes such as FFIT or EuroFIT as part of health-related CSR provision, or interventions targeting other populations, such as women [46] or older adults [47].

The current study has several strengths, one of which is the combination of individual interviews collected in a heterogeneous, cross-country sample and a survey administered to top-tier football clubs in four European countries. In addition, the rigorous approach to analysing cross-country qualitative data in the local language using multiple local research teams increases the trustworthiness of this study. A limitation of this approach, however, is that the first author is unable to check the accuracy of his and others’ interpretations when using translated, descriptive reports, but this was mitigated by other co-authors critically reviewing both the interpretation and integration of local data throughout the analysis process. It is also important to note that this study retrospectively explored motives for adopting EuroFIT in RCT clubs who received full funding and had already delivered the programme, and therefore, may not fully reflect all aspects of decision-making processes compared to clubs who decide not to adopt CSR programmes. Another limitation is the expected selection bias of survey respondents. Given that the survey sought to review existing health-promotion practices in football clubs, it is likely that online respondents from clubs with greater levels of pre-existing commitment and funding to deliver health programmes were more inclined to respond.

## Supporting information

S1 Dataset Anonymized survey data. (XLSX)

S1 File Codebook for qualitative analysis of interviews. (PDF)

S2 File Comparison of motives for adopting EuroFIT by country. (PDF)
