# Supplementary material for: Exploring the provision and motives behind the adoption of health-promotion programmes in professional football clubs across four European countries
Source: PLoS One. 2021 Nov 19;16(11):e0259458. doi: 10.1371/journal.pone.0259458 (PMC8604315; doi:10.1371/journal.pone.0259458)
Supplement: S1 File — (PDF) [file pone.0259458.s002.pdf]

CODEBOOK: MOTIVES FOR ADOPTING EUROFIT

Version 5_FINAL

CODE	DESCRIPTION	EXEMPLARY QUOTE
Club philosophy and ethos	This code refers to any mentions of club ethos, or social responsibility philosophy/vision/strategy that coincided with the aims of EuroFIT, as a motive for joining the program. This includes staff/students doing preliminary explorations to figure out if the program fitted the philosophy or vision of the club	"… there has to be a fit with the clubs' policy – we focus on participation, health, and education. Within these domains, we can choose the projects we like (RCTClub206_NED)"
Doing it for the fans	This code refers to any mentions of the club wanting to do the program for the fans.	"for me it's a no-brainer … it's a men's health project and as I say seventy-five, maybe seventy per cent of our fan base probably fit the criteria (RCTClub103_UK)".
Novelty of program	This refers to any mentions of the novelty of the program, or that the club did not offering similar programs, or any program at all to the target group.	"None of the clubs (in the first division) [have] offered a programme like this to its fans before (RCTClub413_PT)"
Marketing/PR purposes	Refers to any form of marketing or PR purposes that the club stated as a motive for joining EuroFIT. This includes reaching new groups of people, sponsors and PR possibilities	"… the return on the investment is the club image, to have the fans looking at the club as a club that is close to its fan base (RCTClub415_PT)"
Credibility	This refers to mentions of the credibility of an academic research project as a motive for joining and also that the program was based on evidence of FFIT	"… it was really the evidence behind it. We wanted to see what worked, have a go, give it the pilot, and then get that evidence back so then, like I say, when we go to run a larger project and do it on a larger scale, we have that evidence, we know … what does and doesn't work

		(PilotClub102_UK)”
Collaboration with University	This refers to any mentions of the university as a collaborative partner as a reason. This includes reflections on the prospect of doing future projects together with the institution.	“… working with the university as well, as a collaborative partner, [because] it might just be that, from this, we could start other projects together with them (RCTClub312_NOR)”
Belief in the project	This refers to any mentions, actions or reflections that suggests that the club manager or someone at the club believed in the program and that this was an important motive for why the club joined EuroFIT	“…we (foundation managers) have that role where we can say yes to anything we think is good. So yes, that is why this project fit well (RCTClub208_NED)”
Financial support	This refers to any mentions of financial support or guarantees from the project that suggested that the club did not have to pay for anything related to delivery	“… because the club is not able to make big investments, we would do the project as long as it was cost-free (RCTClub414_PT)”
Club capacity	This refers to any mentions of having the staff capacity and/or facilities at the club as a motive for agreeing to run EuroFIT.	“P: But they just wanted to know exactly how it was gonna work, what impact would that have, whether it was an evening based course, or opening the stadium, and the facility use.<br>I: Right.<br>P: Erm, but they were fine, overall, once we gave them the plan of what we were gonna do (RCTClub104_UK)”
Experience with similar projects	This refers to any mentions of previous experience of running social projects at the club as a reason for agreeing to deliver EuroFIT. This includes the club having coaches with relevant competence or experience, but do not refer to staff capacity (HR capacity)	“I think it was easy for us (to join the programme) because we have worked with these social projects before, so then it wasn't so difficult to sell it to us ... but I think perhaps that it might be more difficult (to adopt the programme) if you have not worked with supporters already, or other target groups (RCTClub312_NOR)”

Program package	This refers to the club stating that receiving a full/all-in-one program package at the outset, which was clear and easy to understand as a motive for joining.	"I felt that the training and support that was being offered to staff was very good, because we don't have any health professionals' (PilotClub2_ UK)".
Personal connection	This code refers to mentions of a personal connection to, or the reputation of a delivery partner or a significant person obtaining a similar role as a motive for doing the program	"P: I met [name of person] at a conference in September, just before I'd taken up post here [and] I was aware of the programme because I'd previously worked another [that] football club (PilotClub102_UK)".
Collaboration with partners	This refers to any mention of support from partnering organizations or significant stakeholders, such as business partners, sponsors, supporter clubs as a motive for joining the program.	"There is a market for these types of health-related projects. There are health companies that may have interest in being associated to a physical activity programme … I believe other businesses, like clinics with whom we work, could be interested in being close to people (RCTClub413_PT)"
Generate internal support	This refers to any mentions of using the results of the program as a way to legitimize or generate leverage for doing similar projects at the club, and that this was a motive for wanting to join EuroFIT	"As a foundation, it is quite difficult to show what the effect of your program is [within the club]. With EuroFIT it was the perfect opportunity because a scientific evaluation was linked to it. So that is actually the reason (RCTClub209_NED)"
Club endorsement	This refers to any mentions of the success stories of previous or similar program from other football clubs as a motive for wanting to adopt EuroFIT	"We went to the meeting in England (an information meeting offered to potential clubs interested in delivering EuroFIT) to listen to the experiences of clubs in Scotland, as its inception there, the FFIT (Football Fans in Training) programme. So, based on those experiences we said, yes, this is what we actually should do (RCTClub206_NED)"

Potential expansion of activities	This code refers to any mentions or reflections that addresses ideals, thoughts or wishes related to using elements or activities in the EuroFIT program and expanding these two new target groups not covered by the program, e.g. overweight youth, women, family	I remember that we [thought], in the beginning, when introduced to that fact that it should be this and this target group, overweight [men] and that sort of thing … if this is successful, then perhaps we can offer it to different target groups, especially a project in terms of following overweight youth (Club rep_RCTClub312_NOR).
