# Supplementary material for: Exploring the provision and motives behind the adoption of health-promotion programmes in professional football clubs across four European countries
Source: PLoS One. 2021 Nov 19;16(11):e0259458. doi: 10.1371/journal.pone.0259458 (PMC8604315; doi:10.1371/journal.pone.0259458)
Supplement: S2 File — (PDF) [file pone.0259458.s003.pdf]

Comparison of motives for adopting EuroFIT by country

Code	NOR	PT	NED	UK
Views of the programme				
Programme package			X	X
Potential expansion of activities	X	X		X
Financial support	X	X	X	
Novelty of programme	X	X	X	
Marketing/PR purposes	X	X	X	X
Credibility	X	X	X	X
Club motivations				
Doing it for the fans	X	X	X	X
Generate internal support	X		X	X
Club philosophy and ethos	X	X	X	X
Club capacity	X	X		X
Experience with similar projects	X			X
Belief in the project	X		X	X
Stakeholders and partners				
Club endorsements			X	X
Personal connection				X
Collaboration with partners	X	X	X	
Collaboration with University	X	X		X
